# Static magnetic field and alternating magnetic field cytotoxic effects on triple negative breast cancer cell line (MDA-MB-231)

**DOI:** 10.1007/s12032-025-03098-1

**Published:** 2025-11-20

**Authors:** Heba S. Quenawy, Heba Nafea, Nermin Salah, Rana A. Youness, Noha Mohamed, Magdy M. Ghannam, Nermeen M. Serag

**Affiliations:** 1 Physics Department, Faculty of Basic Science, German University in Cairo, New Cairo City, Cairo Egypt; 2 Biochemistry Department, Faculty of Dentistry, German University in Cairo, New Cairo City, Cairo Egypt; 3 Pharmaceuitical chemistry Department, Faculty of Pharmacy, German University in Cairo, New Cairo City, Cairo Egypt; 4 Department of Molecular Genetics and Biochemistry, Faculty of Biotechnology, German International University, Administrative Capital, Cairo Egypt; 5 Biophysics Department, Faculty of Science, Cairo University, Giza, Egypt; 6 Biochemistry Department, Faculty of Pharmacy, German University in Cairo, New Cairo City, Cairo Egypt

**Keywords:** Static Magnetic Field (SMF), Alternating Magnetic Field (AMF), Triple Negative Breast Cancer (TNBC), Cytotoxic effects

## Abstract

Triple-negative breast cancer (TNBC) is described as the most aggressive subtype of breast cancer. TNBC is characterized by the absence of three receptors commonly found in other breast cancer subtypes: estrogen receptor (ER), progesterone receptor (PR), and human epidermal growth factor receptor 2 (HER2). It has a high metastasis capacity, Poor prognosis, and shows a high incidence among young African women with the BRCA1 gene mutation. Thus, TNBC patients are left with limited treatment options. Herein, the magnetic field is chosen as a non-invasive treatment that eliminates the side effects of other conventional therapeutic options. MDA-MB-231 cells were exposed to two types of magnetic fields: a static magnetic field (SMF) and an alternating magnetic field (AMF), with varying intensities and durations. Both SMF and AMF showed cytotoxic effects; cells showed a decrease in viability to percentages up to 58.14 ± 5.51% after exposure to SMF, and 51.17 ± 3.53 % after exposure to AMF, changes in morphological features, changes in populations in cell cycle stages-accumulation in $G_0$/ $G_1$ phases and decrease of population in S and $G_2M$ phases, and a reduction in the total antioxidant capacity of cells to 74.8 ± 0.009 % after exposure to SMF and 89.97 ± 0.006 % after exposure to AMF due to a cytoskeletal disruption. These findings support the use of magnetic fields as a non-invasive alternative to conventional therapies, with further investigation for clinical applications.

## Introduction

As the most prevalent form of cancer among females, breast cancer (BC) stands as the second leading cause of cancer-related deaths globally [1, 2]. This situation emphasizes the critical need for innovative and effective treatment strategies. An Egyptian systematic review and meta-analysis revealed that BC is the most common cancer, accounting for 42% of all cancer cases in females, with the majority presenting in advanced stages [3].

Breast cancers are classified into three main subtypes based on receptor status, which guides targeted treatment approaches [4]. Hormone Receptor-Positive (ER/PR-Positive), characterized by the presence of estrogen receptors (ER) and/or progesterone receptors (PR). HER2-positive (with or without ER/PR positivity) is defined by the overexpression of the Human epidermal growth factor receptor 2 protein, which promotes cancer cell growth. Triple-negative breast cancer (TNBC) lacks ER, PR, and HER2 expression. ER/PR-positive breast cancers can be treated by hormonal therapies such as tamoxifen or aromatase inhibitors, while HER2-positive breast cancers respond well to targeted therapies like trastuzumab (Herceptin) [5]. However, TNBC does not respond to these treatments due to the lack of the three types of receptors found regularly in breast cancer cells (ER, PR, and HER2), leaving chemotherapy as the primary systemic therapy.

TNBC is one of the most aggressive subtypes of invasive breast cancer and represents 10–15 % of all breast cancer types. It tends to grow and spread faster than other breast cancers and has a higher metastasis potential, especially to the lungs and brain, lower survival rates, and a higher risk of recurrence [6]. The absence of receptors makes TNBC treatment challenging, and this encourages further research into new treatment options.

Current popular cancer treatments, such as surgery, radiotherapy, immunotherapy, and chemotherapy, while effective to some extent, have significant limitations that prevent them from being fully efficient in treating aggressive cancer cells [7]. These limitations include the inability to avoid metastasis [8], severe side effects on healthy tissues, and the risk of drug resistance [9]. This highlights the urgent need for new, more effective treatments.

The potential of using magnetic fields (MFs) as a non-invasive cancer treatment could be a significant milestone in cancer therapy. This approach could revolutionize cancer treatment, eliminating the side effects of other treatment types. Compared to traditional therapies, MFs are non-invasive, safe, highly efficient, inexpensive, and free from the risk of infection or scarring [10].

MF is a physical field that transmits energy to the medium it passes through and exerts a magnetic force on charged particles inside it. Magnetic fields are categorized into two types: uniform and non-uniform. A uniform magnetic field maintains a consistent or nearly consistent intensity throughout a given space. In contrast, if the field’s intensity varies across different regions, it is referred to as a non-uniform magnetic field [11].

Previous work focused on adopting uniform magnetic fields to ensure a consistent, controlled, and homogeneous effect on cancer cells during magnetic field-based treatments. This approach enhances treatment precision, maximizes therapeutic efficacy, and minimizes unintended variations in cellular response. Uniform magnetic fields can be divided into static (SMF) or dynamic (DMF). SMF has a constant magnitude and direction that can be generated by permanent magnets or coils with unidirectional currents. We can classify SMFs as weak (< 1 mT), moderate (1 mT - 1 T), strong (1 - 5 T), and ultrastrong (> 5 T) [12].

DMFs are fields whose amplitudes and polarities vary with time. Depending on the mechanism of magnetic field production, DMF can be categorized as an alternating magnetic field (AMF) generated by an electromagnetic coil with a current of a particular frequency, a pulsed magnetic field (PMF) produced by an electromagnetic coil with a pulsed current, rotating magnetic field (RMF) produced by a magnet with regular motion or rotation, and geomagnetic field (GMF) produced by earth and ionosphere [13].

Exposure of cancer cells to a magnetic field produces intriguing biological effects. Magnetic fields have different interaction mechanisms with cancer cells, leading to cell death. Zablotskii et al. found that the magnetic susceptibility of tumor cells differed owing to their different cellular contents. Therefore, the biological effects of MF on tumor cells depend on various MF parameters, such as intensity, frequency, and exposure duration, as well as the type of tumor cell [14]. The biological effects of magnetic treatments are mainly due to the impact of the treatment on the medium properties, physicochemical properties of cell membranes, the structure of biological macromolecules, and free radicals [11].

Preliminary studies have shown that MFs can affect tumor cell morphology, membrane structure, cell metabolism, growth, adhesion, immune response, and microcirculation [15]. The disruption of free radical metabolism, specifically that of ROS (reactive oxygen species) and RNS (reactive nitrogen species), and an elevation in the nuclei of cells have higher magnetic moments ($\upmu$). Thus, making them highly sensitive to magnetic force. This potential to disrupt cancer cell growth and proliferation is an exciting area of research that holds promise for future cancer treatments.

The primary cause of cell changes after incubation in external SMF is disruption of free radicals, for example, ROS (reactive oxygen species) and RNS (reactive nitrogen species) metabolism, and elevation of their concentration. Breast cancer cells exposed to SMF showed increased ROS levels [16]. Free radicals are incredibly oxidative and, as a result, damage ion channels, leading to changes in cell morphology and expression of different genes, and damaging DNA, proteins, and lipids. This leads to changes in apoptosis and proliferation [12].

In addition, MF induces Joule’s heating that expands tumor blood vessels, leading to excessive oxygen entering the cell, which hinders cancer cells’ survival [17]. Moreover, MF can disturb the vascular epithelial growth factor (VEGF) level, decreasing the growth and dissemination of cancer to other body parts [18].

The maintenance of cell morphology is crucial for cellular survival, as it ensures proper function, structural integrity, and communication. Disrupting cell morphology through MF exposure can destabilize the cytoskeleton, impair cellular processes, and ultimately trigger cancer cell death by inducing mechanical stress, apoptosis, or necrosis. Exposure to MF leads to the formation of bubbles on the cell membrane [19], shrunk in cell bodies, and cytoplasmic protrusions [20]. The phospholipid bilayer and proteins that make up the cell membrane serve as a barrier to keep the intracellular environment stable. Furthermore, the cell membrane functions as a selective permeability interface, enabling the creation and maintenance of ion concentration differences that result in potential differences. These differences are vital for the survival and proliferation of cells. MFs act on several ion channels ($\textrm{Ca}^{+2}$, $Na^+$, etc.) to alter potential differences. Disrupting these potential differences by MF exposure affects cell proliferation [21]. MF can promote membrane permeability, thus disturbing the cell’s physiological balance. The Lorentz force induced by the MF affects the moving charges, which in turn affects the permeability of the membrane, thus disrupting the selective transmissibility of ion channels [22]. Moreover, MF increases both the number and size of membrane pores, leading to enhanced cell membrane permeability [23].

Additionally, after MF exposure, cytoplasmic vacuoles of different sizes appear in cancer cells, indicating cellular stress, damage, and potential cell death [24]. The key metabolic organelles, which are crucial for tumor development, such as mitochondria, are highly affected by MF exposure. This effect is attributed to the disruption of mitochondrial ultrastructure, resulting in a decrease in ATP synthesis and an increase in ROS generation [15]. In addition, the Diamagnetic nucleus is among the targets of interaction with the magnetic field due to the charges present on it [25]. MF induces DNA damage in tumor cells, thereby inhibiting their proliferation by breaking DNA strands and reducing the nucleocytoplasmic ratio (N/P), which in turn inhibits DNA replication and ultimately cell mitosis. This effect may be associated with the Lorentz force’s ability to alter the low-energy hydrogen bond in DNA [26].

It is worth mentioning that using magnetic fields to treat breast cancer, while promising, faces several significant limitations. One major challenge is the potential effect on surrounding tissues; this is particularly concerning in breast cancer treatment, where nearby tissues, such as the heart and lungs, are highly sensitive. Additionally, the mechanisms by which magnetic fields affect cellular processes are not fully understood, making it challenging to optimize treatment parameters, such as intensity, frequency, and duration of exposure. Another limitation is the limited penetration depth of magnetic fields, especially in the case of static or low-frequency fields. Breast tumors located deep within the tissue may not receive adequate exposure to the magnetic field, reducing the treatment’s efficacy. Finally, there is a lack of large-scale clinical trials to validate the safety and effectiveness of magnetic field therapy for breast cancer. While preclinical studies show potential, more robust evidence is needed to establish magnetic fields as a reliable and standardized treatment option in clinical practice.

In the present study, two types of magnetic fields, static magnetic field (SMF) and alternating magnetic field (AMF), were selected with varying intensities. Triple-negative breast cancer (TNBC) cells were exposed to both fields for different time intervals to determine the optimal intensity and exposure period. Cell viability was determined using the MDA-MB-231 cell line in an MTT assay under various MF exposure conditions. Preliminary investigations were conducted to explore possible underlying mechanisms of action for observed cytotoxicity.

## Materials and methods

### Exposure system

**Fig. 1:**
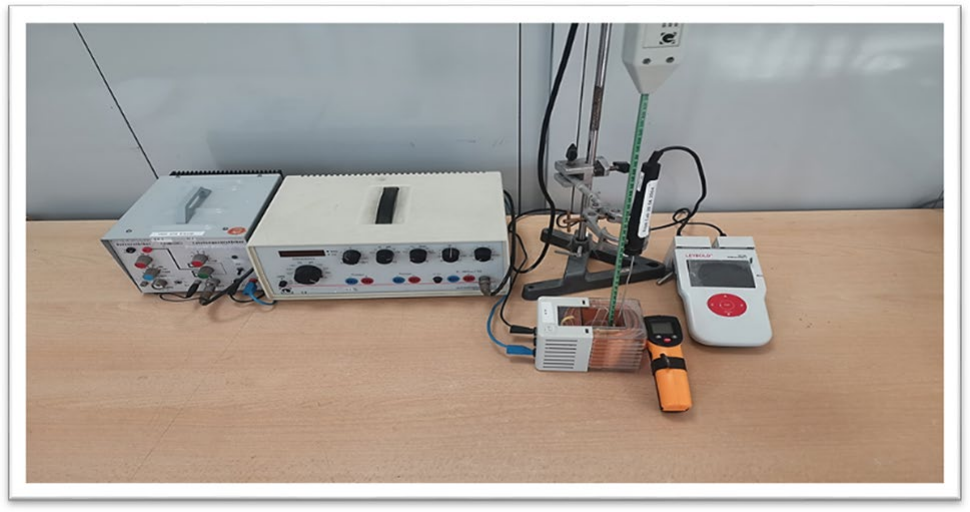
Magnetic field exposure system

SMF and 100 kHz (AMF) were generated by a coil (Leybold 562 15), N = 1000 turns, L = 36 mH, R = 9.5 $\Omega$, and Imax = 1.25A. The coil is connected to the power generator (Leybold 522 63), 0–30 Vp-p/3 A, and a Power Amplifier (up to ± 12 V). Magnetic field strength is measured by an axial B-probe (Leybold 516 61) connected to Mobile Cassy 2 (Leybold 524 005 W2). A NiCr-Ni thermocouple (Leybold 666 193) connected to Mobile Cassy through a temperature box (Leybold 524 045) recorded the vacuum temperature inside the coil. The temperature of the cells was recorded before and after exposure by an infrared thermometer. See Figure 1. Cells were exposed to SMF and AMF strengths of 5, 10, and 15 mT for 10, 20, 30, 60, and 90 minutes.

### Cell culture

TNBC cell line MDA-MB-231 was maintained in Dulbecco’s modified Eagle’s medium (DMEM; Lonza, Basel, Switzerland) supplemented with 4.5 g/L glucose, 4 mmol/L L-glutamine, 10% fetal bovine serum (FBS), and MycoZap (1:500; Lonza) at 37$^{\circ }$C in a 5% $CO_2$ atmosphere. The cells were transferred upon reaching 70–80% confluency to a 3.5 mm petri dish as described in [27]. After exposure to MFs of different intensities for different time durations, cells were maintained at 37$^{\circ }$C and 5% $CO_2$ for 24 hours. At the end of incubation, the following tests were conducted on harvested cells.

### Cellular viability test

The cytotoxicity of the magnetic field is measured by 3- (4, 5-dimethylthiazol- 2-yl) −2, 5-diphenyl tetrazolium bromide (MTT assay). Cells were transferred to a 96-well plate, and 10,000 MDA-MB-231 cells were seeded in 200 µl of media. Forty-eight hours after exposure, the media was removed, and 20 µl of working solution was added to each well. After 6 hours, the resulting formazan crystals were solubilized in 200 µl lysis buffer, and absorbance was measured as described in [27–29]. The MTT data were compared to the control data obtained for the non-exposed cells and blank reagent.

### Morphological assessment

The MDA-MB-231 cultured cells were stained with hematoxylin and eosin (H&E) for morphological assessment. This process began by fixing the cells with 10% formalin for 10–15 minutes at room temperature. After fixation, Cells were washed with phosphate-buffered saline (PBS) to remove residual formalin. Next, Cells were stained with hematoxylin solution for 5–10 minutes, then rinsed with distilled water. The cells were briefly dipped in an acidic alcohol solution (1% hydrochloric acid in 70% ethanol) and then rinsed with distilled water. Eosin was applied for 1–2 minutes to counterstain the cytoplasm, followed by a final wash in distilled water. Finally, the cells were dehydrated by passing them through graded alcohols, cleared with xylene, and mounted with a coverslip using a permanent mounting medium for microscopic examination. The cells were morphologically examined by a Labomed inverted microscope (Labomed, USA), Vega Digital Camera live-cell imaging software (Labomed, Los Angeles, USA), and LC-6 USB3.0 Colorful CMOS Digital Cameras (Labomed, Los Angeles, USA).

### Measuring the total antioxidant capacity in cultured cells

The Total Antioxidant Capacity (TAC) was measured in cultured cells using the following protocol. First, the cultured cells were harvested by scraping and then washed with cold phosphate-buffered saline (PBS) to remove any remaining media and extraneous substances. After washing, the cell pellet was collected by centrifugation at 1500 rpm for 5 minutes. The cells were lysed by adding an appropriate lysis buffer, such as RIPA buffer, supplemented with protease inhibitors. Ensure thorough cell lysis by incubating the lysate on ice for 20–30 minutes, followed by sonication if necessary. The cells were lysed again at 12,000 rpm for 10 minutes to remove cell debris and collect the supernatant for further analysis. Prieto et al.’s method assessed total antioxidant activity (TAC) [30]. The absorbance was determined at 695 nm against a blank after cooling the reaction at room temperature. Total antioxidant capacity was measured using the Total Antioxidant Capacity (T-AOC) Colorimetric Assay Kit (cat no: E-BC-K136-M, Elabscience Biotechnology, USA)

### Cell cycle assay by flow cytometry

Cells were harvested 24 hours after exposure to MF. Following scraping, the cells were fixed with cold methanol, stained with Vybrant DyeCycle Violet stain (Invitrogen, cat. no. V35003), and examined by flow cytometry to visualize the DNA content distribution during different cell cycle stages. One mL of cell suspension in complete media at a cell concentration of $1 * 10^6$ cells/mL was added to 1 µL of Vybrant$\circledR$ DyeCycle$^{{\textrm{TM}}}$ Violet stain and mixed well. The final stain concentration became 5 µM. The stained cells were incubated at $37^0C$ for 30 minutes, and protected from light until acquisition–finally, the Beckman Coulter Navios EX software: SM: BE14548 software version: Navios EX. (Beckman Coulter) was used to analyze the data with an excitation wavelength of 405 nm and an emission wavelength of 440 nm. Analysis of the population indicates the following distribution: apoptotic $sub-G_1$ cells, $G_0$/phase, S phase, and $G_2M$ phase [31].

### Statistical analysis

Statistical analysis was carried out using GraphPad Prism version 8.0.1 (GraphPad Software, San Diego, CA, USA). Results are expressed as mean ± standard deviation (SD). A one-way ANOVA followed by a Student’s t-test was used to assess statistical differences between the experimental groups and the control, as well as pairwise comparisons. A P value < 0.05 was considered statistically significant. All experiments were performed in triplicate (n = 3).

## Results

### Cell viability

**Fig. 2:**
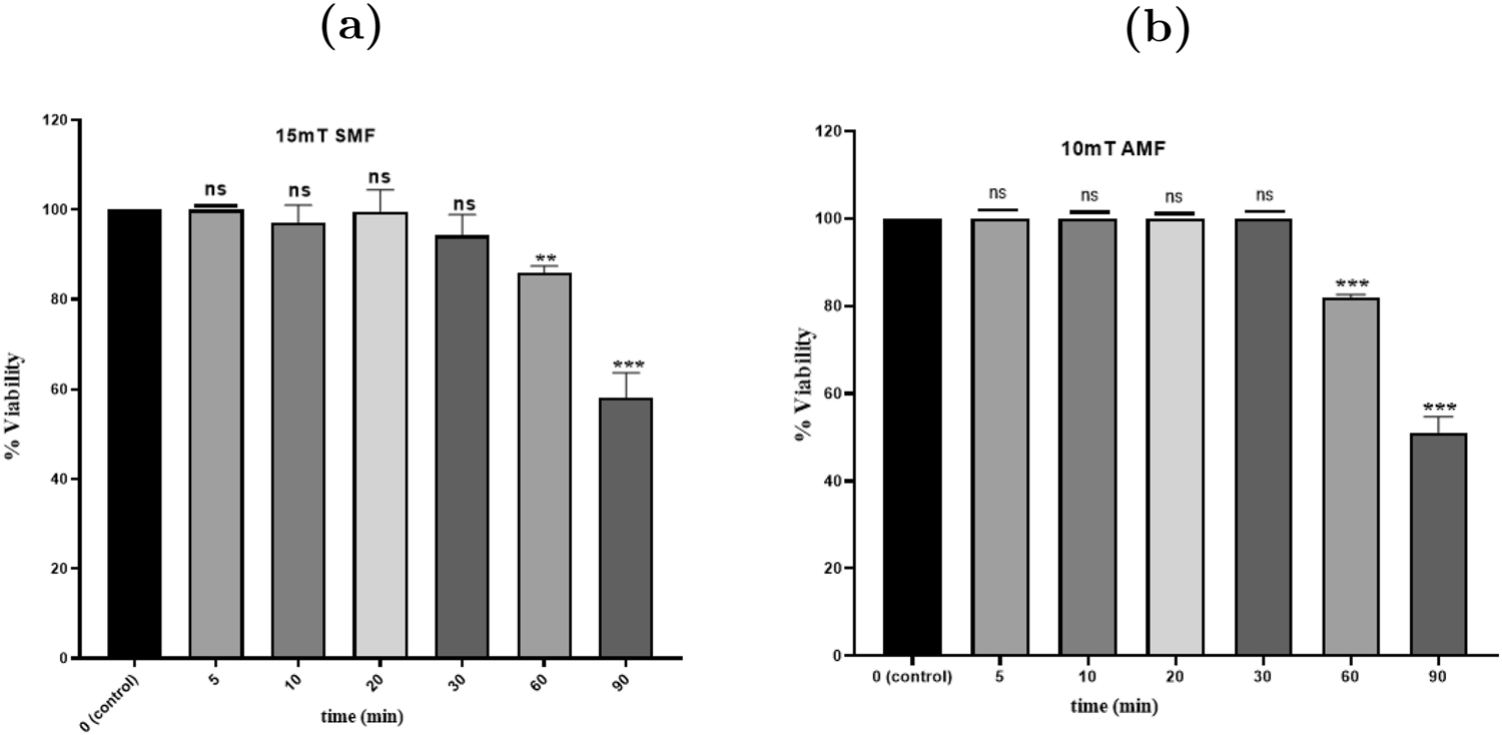
Cellular viability of MDA-MB-231 cells following exposure to (**a**) 15 mT SMF and (**b**) 10 mT AMF for durations of 5, 10, 20, 30, 60, and 90 minutes. Statistical significance is indicated as follows: P < 0.05(*), P < 0.01 (**), P < 0.001 (***), P > 0.05 (ns)

Cells were exposed to SMF intensities of 5, 10, and 15 mT for 5, 10, 20, 30, 60, and 90 minutes. MTT results showed that 5 and 10 mT were non-significant for all exposure periods compared to the control. Meanwhile, the 15-mT field showed a significant decrease in viability only for exposure periods of 60 & 90 minutes. The percentage of viable cells decreased to 87.03 ± 1.44 % (P < 0.05) and 58.14 ± 5.51% (P < 0.05), respectively (Figure 2a).

Cells were also exposed to AMF for the same intensities and periods. MTT results for AMF exposure showed no significance in all periods for 5 & 15 mT intensities. While 10 mT showed only a significant decrease in viability for exposure periods of 60 & 90 minutes. The percentage of viable cells decreased to 81.87 ± 0.75 % (P < 0.05) and 51.17 ± 3.53 % (P < 0.05), respectively (Figure 2b).

**Fig. 3:**
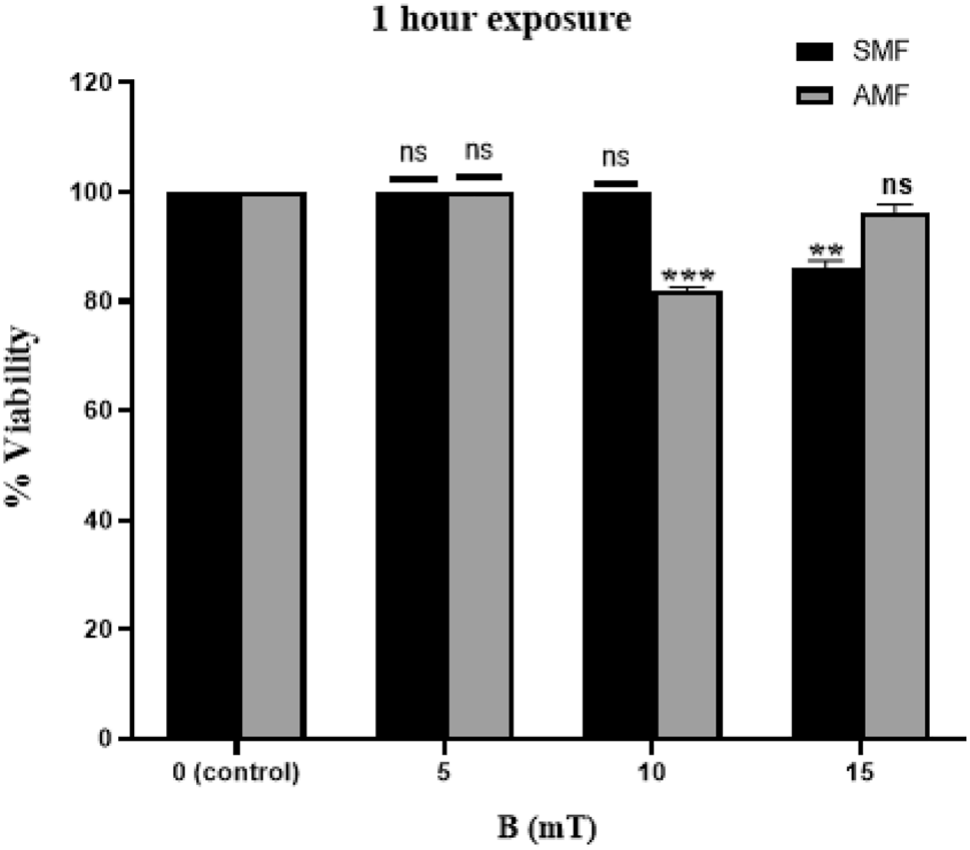
Cellular viability of MDA-MB-231 cells following exposure to Static Magnetic Field (SMF) and Alternating Magnetic Field (AMF) at intensities of 5, 10, and 15 mT for 60 minutes. Statistical significance is indicated as follows: P < 0.05 (*), P < 0.01 (**), P < 0.001 (***), P > 0.05 (ns)

The graph trend line indicates a progressive decrease in the percentage of viable cells with increasing exposure time, with a minimum exposure time of 60 minutes (Figure 3). Based on this observation, two conditions showing significant effects - 15 mT SMF and 10 mT AMF applied for 60 minutes - were selected for further investigation.

### Temperature-Magnetic field stability

**Fig. 4:**
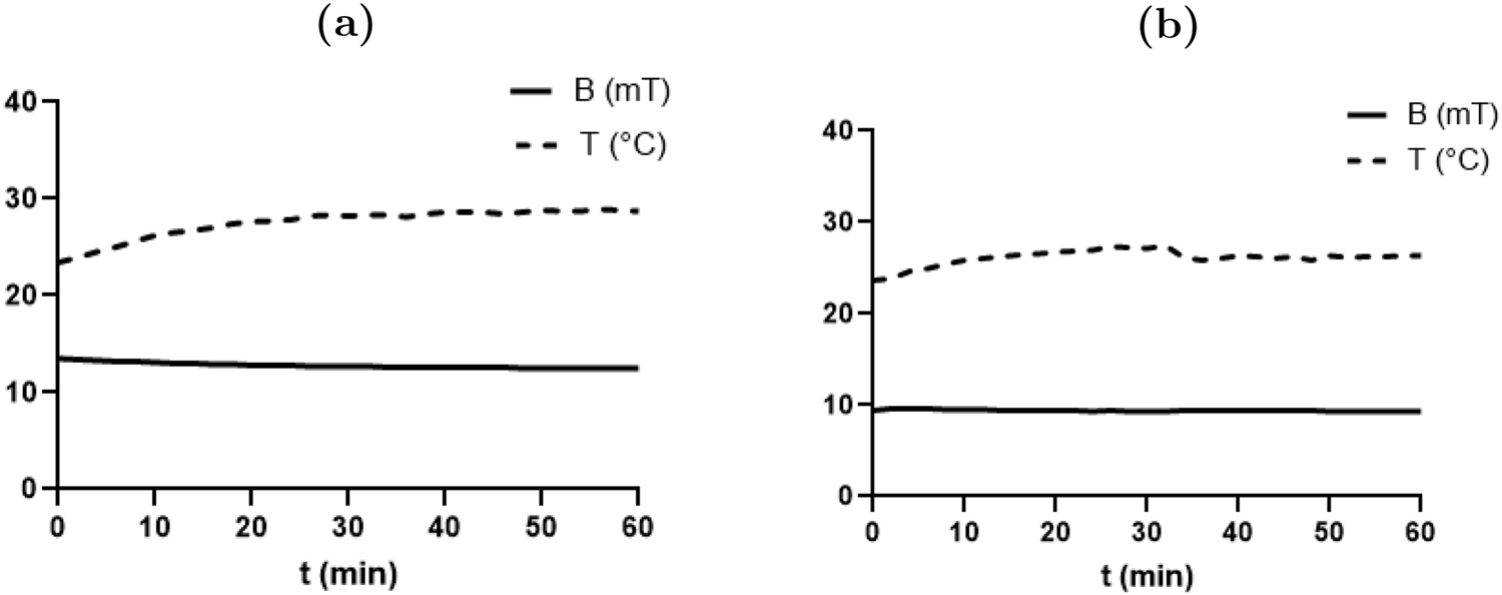
Temperature and magnetic field stability over a 60-minute exposure period. (**a**) SMF, 15 mT; (**b**) AMF, 10 mT

Both temperature and magnetic field strength were continuously monitored during the entire exposure period (60 minutes). The data confirmed consistent temperature and field intensity. (Figure 4).

### Morphological assessment

**Fig. 5:**
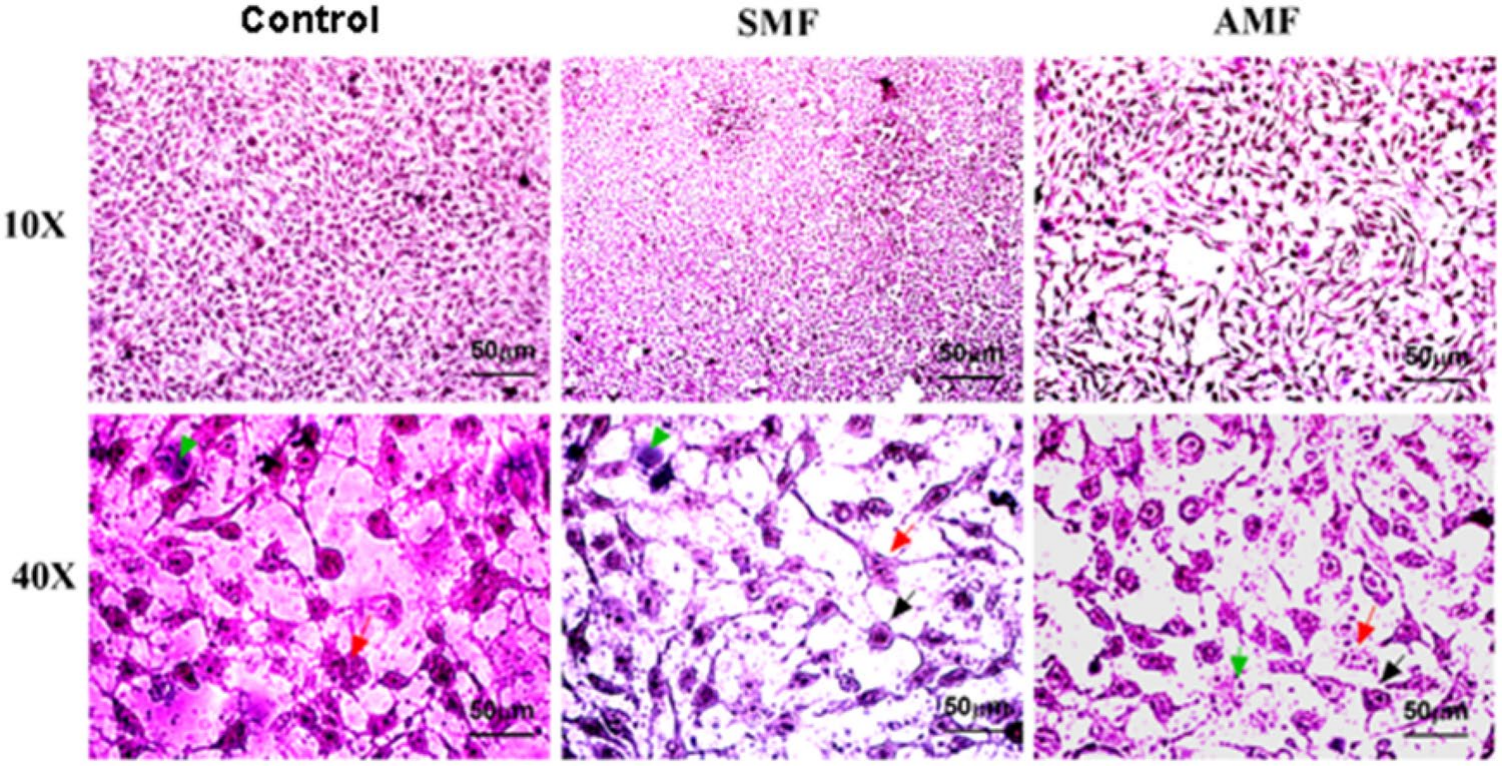
Morphological changes in MDA-MB-231 cells following exposure to magnetic fields. Images of control cells and cells exposed to SMF and AMF were captured 24 hours post-exposure. Images were taken using a Labomed inverted microscope (Labomed, USA) equipped with a Vega Digital Camera at 10$\times$ and 40$\times$ magnifications. Scale bar: 50 $\upmu$m

MDA-MB-231 cells were exposed to 15 mT SMF and 10 mT AMF for 60 minutes to determine the type of cell death induced by the magnetic field. Cells were then stained with hematoxylin and eosin (H&E) for morphological assessment. Microscopic analysis of H&E-stained MDA-MB-231 cancer cell samples revealed significant morphological differences between untreated control cells and cells exposed to SMF and AMF, as shown in Figure 5.

The most distinguished alterations in nuclear morphology appeared as nuclear fragments (red arrows) or condensations (black arrows). Other morphological changes, such as membrane blebbing or protrusions, cytoplasmic vacuolations, cell shrinkage, and apoptotic nuclear changes (green arrows), also appeared.

### Measuring the total antioxidant capacity in cultured cells

**Fig. 6:**
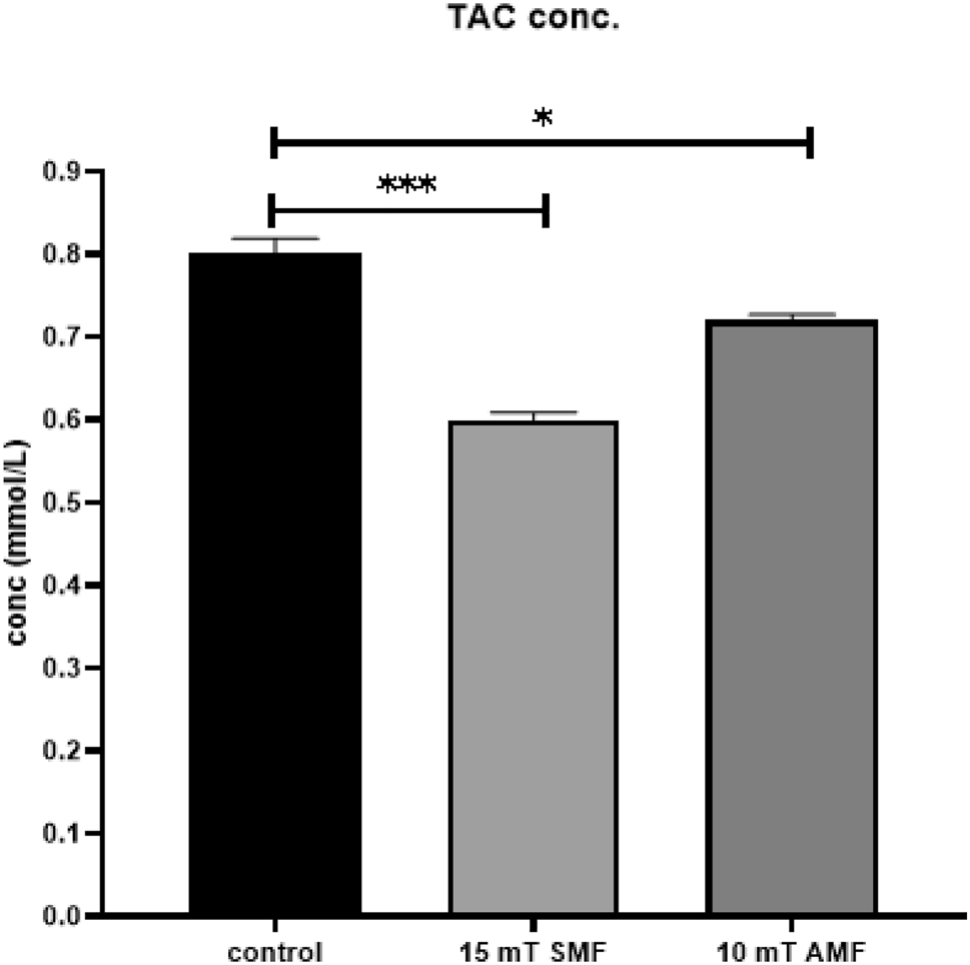
Antioxidant concentrations measured in control cells and cells exposed to 15 mT SMF and 10 mT AMF for 60 minutes. Statistical significance is indicated as follows: P < 0.05 (*), P < 0.001 (***),(n=3)

Cells were exposed to 15 mT SMF and 10 mT AMF for 60 minutes to assess the antioxidant status of TNBC. Then, the total antioxidant capacity of the samples was measured. Results representing the optical density and corresponding concentrations of antioxidants for three replicate samples (n=3) are described in Table 1. It was found that exposure to SMF and AMF decreased the total concentration of antioxidants in TNBC to 74.8 ± 0.009 % (P < 0.001) and 89.97 ± 0.006 % (P < 0.05), respectively. A graphical representation of the changes in total concentration of antioxidants following exposure to SMF and AMF is shown in Figure 6

**Table 1:** Average effect of static magnetic field (SMF) and alternating magnetic field (AMF) on the total antioxidant capacity of MDA-MB-231 cells after 60 minutes of exposure (n=3)

Experimental group	OD$^{1}$at 695 nm	Concentration (mmol/L)
Control	0.2427 ± 0.006	0.8008 ± 0.0175
SMF-exposed	0.1817 ± 0.0029	0.5995 ± 0.0115
AMF-exposed	0.2183 ± 0.0025	0.7205 ± 0.0075

$^{1}$OD: optic density

### Cell cycle assay by flow cytometry

To study the mechanisms of the cytotoxic effects of the MF on TNBC, we exposed MDA-MB-231 cells to 15 mT SMF and 10 mT AMF for 60 minutes. Then, we used flow cytometry to test whether the cell cycle distribution was affected. Results from flow cytometry for three replicate samples (n=3) are presented in Table 2. The distribution during the four phases, $G_0$, $G_1$, S, and $G_2M$, is shown for the two types of magnetic field.

**Table 2:** Distribution of cell population during different phases of the cell cycle for control cells and cells exposed to SMF and AMF for 60 minutes (n=3)

Cell cycle phase	Control (%)	SMF-exposed (%)	AMF-exposed (%)
$G_0$	0.0 ± 0.0	4.3 ± 0.0	13.6 ± 0.0
$G_1$	0.2 ± 0.0	15.73 ± 0.06	32.07 ± 1.10
S	49.8 ± 1.93	49.47 ± 0.35	37.83 ± 1.76
$G_2M$	47.87 ± 0.71	30.13 ± 0.06	16.47 ± 0.06

**Fig. 7:**
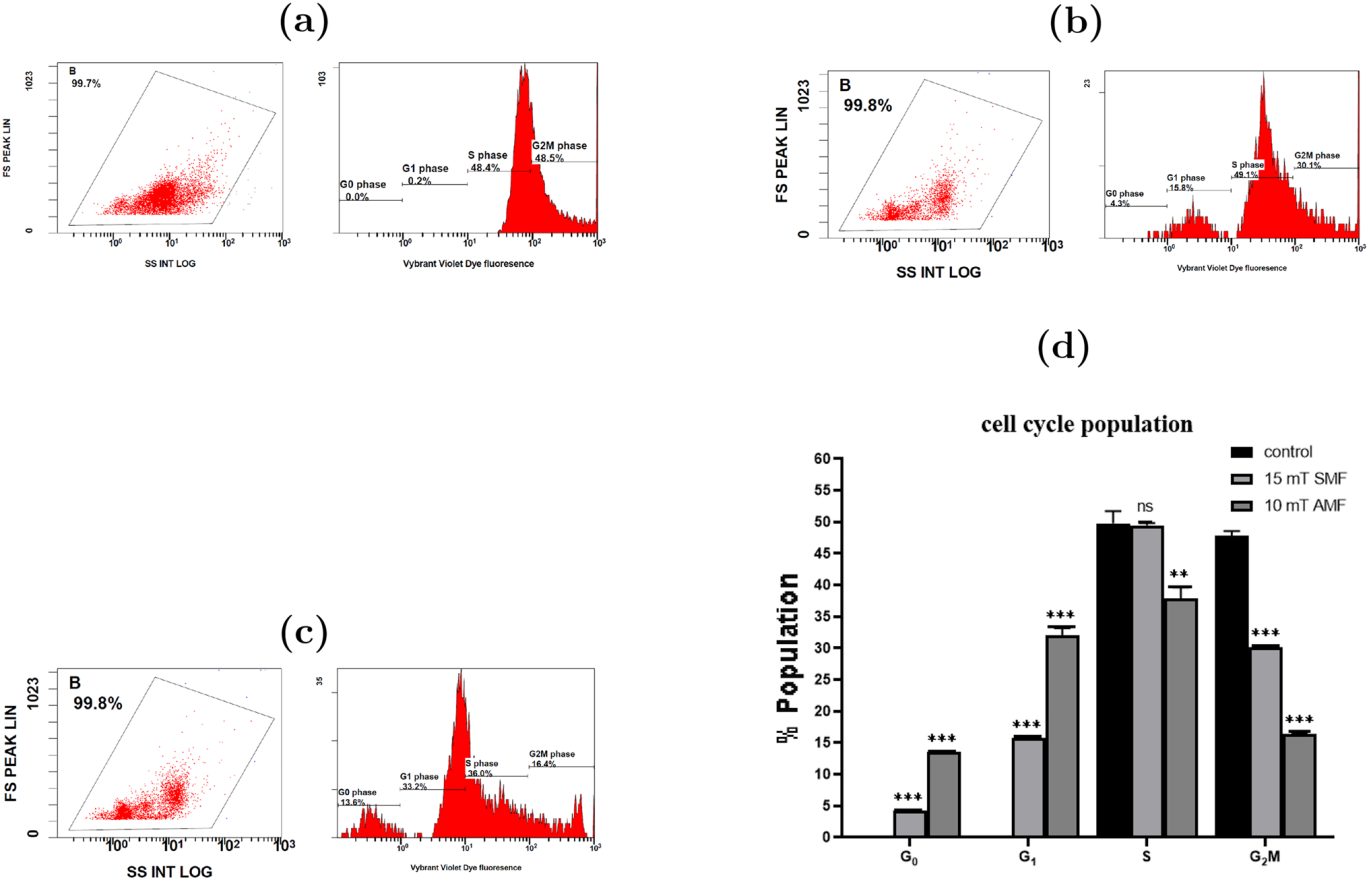
Flow cytometric analysis of cell cycle distribution in MDA-MB-231 cells. DNA content histogram for (**a**) control, (**b**) exposed to 15 mT SMF, and (**c**) exposed to 10 mT AMF. Showing cell populations in $G_0$, $G_1$, S, and $G_2M$ phases. Peaks represent cell populations in distinct phases of the cell cycle. (**d**) Graphical representation of cell population percentages across $G_0$, $G_1$, S, and $G_2M$ phases (n = 3). Statistical significance is indicated as follows: P < 0.05 (*), P < 0.01 (**), P < 0.001 (***), P > 0.05 (ns)

Results showed an accumulation of cells in the $G_0$/$G_1$ phase for both SMF and AMF, as well as a decrease in population in both S and $G_2M$ phases compared to the control phase (Figure 7).

In control (untreated) cells, the population was predominantly distributed between the S and $G_2M$ phases, indicating high proliferative activity. Exposure to 15 mT SMF and 10 mT AMF increased populations of $G_0$ phase from 0.0 ± 0.0 % to 4.3 ± 0.0 % (P < 0.001) and 13.6 ± 0.0 % (P < 0.001), respectively. The $G_1$ population rose from 0.2 ± 0.0 % in control to 15.73 ± 0.06 % (P < 0.001) (SMF) and 32.07 ± 1.10 % (P < 0.001) (AMF).

In contrast, the proportion of cells in the S phase decreased from 49.8 ± 1.93 % (control) to 49.47 ± 0.35 % (not significant) (SMF) and 37.83 ± 1.76 % (P < 0.01) (AMF), while the $G_2M$ phase declined from 47.87 ± 0.71 % (control) to 30.13 ± 0.06 % (P < 0.001) (SMF) and 16.47 ± 0.06% (P < 0.001) (AMF).

## Discussion

Recently, TNBC has attracted significant attention due to its aggressive behavior and lack of effective treatment methods [32]. Many alternative treatment methods are being explored to affect it and minimize side effects on healthy tissues. Magnetic field has been chosen as a non-invasive alternative treatment for cancer. It showed good potential in inhibiting cancer cell growth. The cytotoxicity of MFs on MDA-MB-231 cells depends on the parameters of the field, including intensity, frequency, and exposure duration.

In the present study, the exposure system was evaluated using varying intensities and exposure durations for both static magnetic fields (SMF) and alternating magnetic fields (AMF) to identify the threshold conditions at which significant biological effects first emerge. Notably, a previous study demonstrated that this system produces significant effects at field intensities of 5, 10, and 15 mT, providing a basis for selecting the tested parameters [33].

Exposure to 15 mT SMF for a minimum 60-minute duration showed a significant decrease in cellular viability, ranging from 12.97% to 41.86%. Decrease in viability can be attributed to the ability of SMF to induce apoptosis [34–36]. The effect of SMF on reducing the growth rate of cancer cells differs from one cell type to another. SMF exposure increased the apoptotic cell rates in MCF-7 and MDA-MB-231 cell lines [37]. Several hypotheses can describe the fatal effect of SMF on cancer cells. Among those are the effects of suppression of angiogenesis, blood supply blockage, and cell death due to apoptosis [38]. Additionally, the increase in oxygen free radicals alters the proliferation rate and viability of cancer cells [39].

Exposure to 10 mT AMF for a minimum duration of 60 minutes resulted in a decrease in viability ranging from 18.13% to 48.83%. AMF exposure showed antiproliferative and apoptotic activity in breast cancer cells [40–43]. Elevation in ROS inhibits cancer cell growth and therefore promotes mitochondrial biogenesis [44]. The stability of the magnetic field throughout the exposure period excludes the influence of field fluctuations on the observed biological effects. Moreover, maintaining the temperature consistently below the hyperthermia threshold (42$^{\circ }$C) ensures that the cytotoxic effects are not a result of thermal damage, thereby supporting a non-thermal mechanism of action. The most likely explanation for the present results is that MFs affect para- and diamagnetic structures in cells, such as the nucleus and mitochondria. Thus, it affects cells’ viability.

The significant reduction in viability at 10mT AMF exposure, but not at 5mT or 15mT, aligns with the intensity-window phenomenon, where only specific intensity-time combinations evoke a notable biological response. The interaction of AMFs with biological systems is nonlinear, and a particular window of field intensity and exposure duration can produce maximum biological effects. Lower or higher intensities may fall outside this effective range [45]. This response is believed to result from a resonance-like coupling of AMFs with specific cellular components, including ion channels, the cytoskeleton, and radical-pair mechanisms, which can lead to decreased cellular viability. Additionally, field uniformity is crucial for ensuring adequate exposure. At higher intensities, non-uniformities within the coil–caused by waveform distortion or localized heating–may reduce biological coupling. Conversely, 5 mT reflects insufficient field strength. Recognizing and accounting for this intensity-window effect is essential for optimizing magnetic field exposure parameters in therapeutic applications.

One of the possible biological effects of MFs on cancer cells is altering cell morphology. Several studies showed the effect of MFs on cancer cell morphology [19, 20]. Results obtained in our study revealed that exposure to 15 mT SMF and 10 mT AMF for 60 minutes resulted in alterations in nuclear morphology, including nuclear fragments or condensations, as well as membrane blebbing and cytoplasmic vacuolation. Exposure to MFs affects the metabolic activity of cancer cells and their cellular structure, causing DNA damage and enlarged vacuoles inside the cytoplasm [24, 26]

The cell cycle plays a crucial role in regulating cell proliferation, and any disruption in this process can inhibit cell growth or trigger apoptosis. Exposure to MFs has been reported to interfere with normal cell cycle progression, thereby preventing tumor cells from completing the $G_2$ phase and initiating the M phase, ultimately inhibiting proliferation [15]. In breast cancer cells, several studies have demonstrated that MF exposure can alter cell cycle dynamics, such as increasing the proportion of cells in the $G_2M$ phase through the downregulation of Cyclin B1 expression [46, 47].

In the present study, flow cytometry analysis revealed a significant increase in the $G_0$/$G_1$ cell population following exposure to both SMF and AMF, indicating cell cycle arrest or delayed progression. This finding suggests the potential apoptotic effect of MFs, supported by the observed reduction in cell populations in the S and $G_2M$ phases, indicating inhibition of DNA synthesis and blockage of mitotic entry. Such alterations are likely linked to a decrease in the expression of Cyclin D1, a key regulator of the $G_0$/$G_1$ transition. These observations are consistent with previous reports, which show that MF exposure can induce cell cycle arrest at the $G_0$–$G_1$ phase while reducing the proportion of cells in the S and $G_2M$ phases [48].

MF exposure causes oxidative stress in cancer cells. The present study’s data revealed a remarkable decrease in the total antioxidant capacity in TNBC cells after 60 minutes of exposure to 15 mT SMF and 10 mT AMF, with percentages of 25.2% and 10.03 %, respectively. A decreased antioxidant capacity in cancer cells reduces their ability to balance oxidative stress and leads to cell death[49]. The sensitivity of cancer cells to treatments is inversely proportional to their total antioxidant capacity (i.e., low antioxidant capacity indicates high sensitivity to treatment) [50].

## Conclusion

It has been demonstrated that the biological effects of MFs depend on field parameters (intensity, frequency, etc.), exposure duration, and the specific cell type. Our experiment setup significantly affected TNBC cells in exposures to 15 mT SMF and 10 mT AMF for 60 minutes. The impact of MFs on cancer cells primarily focuses on cell morphology, cell membrane structure, and the cell cycle. It is believed that MFs affect the cytoskeleton of cells. Further studies are needed to understand better the cytotoxic effects of different types and intensities of magnetic fields on cancer cells. One limitation of this study was the small coil size, which constrained the selection of sample plate formats and limited the system’s applicability to larger sample volumes. Future work should optimize the coil design to accommodate a broader range of experimental configurations.

## Data Availability

The datasets generated during and/or analysed during the current study are available from the corresponding author on reasonable request.
